# Adverse effects during the oral glucose tolerance test in post-bariatric surgery patients

**DOI:** 10.1590/2359-3997000000149

**Published:** 2016-02-11

**Authors:** Heliana Fernanda de Albuquerque Andrade, William Pedrosa, Maria de Fátima Haueisen Sander Diniz, Valéria Maria Azeredo Passos

**Affiliations:** 1 Departamento de Clínica Médica Faculdade de Medicina da Universidade Federal de Minas Gerais UFMG Belo Horizonte MG Brasil Departamento de Clínica Médica, Programa de Pós-Graduação em Ciências Aplicadas à Saúde do Adulto, Faculdade de Medicina da Universidade Federal de Minas Gerais (UFMG), Belo Horizonte, MG, Brasil

**Keywords:** Oral glucose tolerance test, hypoglycemia, bariatric surgery

## Abstract

**Objective:**

: The oral glucose tolerance test (OGTT) is used in the screening of gestational diabetes, in diagnosis of type 2 diabetes in conjunction with fasting blood glucose and glycated hemoglobin. The aim of this study was to examine the incidence and risk factors of adverse effects of OGTT in patients who underwent bariatric surgery, in addition to proposing standardization for ordering the OGTT in these patients.

**Subjects and methods:**

: This study assessed the incidence of adverse effects in 128 post-bariatric surgery patients who underwent the OGTT. Descriptive and logistic regression analysis were performed, the dependent variables were defined as the presence of signs (tremor, profuse sweating, tachycardia), symptoms (nausea, diarrhea, dizziness, weakness), and hypoglycemia (blood glucose ≤ 50 mg/dL).

**Results:**

: One hundred and seventeen participants (91.4%) were female; 38 (29.7%) participants were pregnant. High incidence (64.8%) of adverse effects was observed: nausea (38.4%), dizziness (30.5%), weakness (25.8%), diarrhea (23.4%), hypoglycemia (14.8%), tachycardia (14.1%), tremor (13.3%), profuse sweating (12.5%) and one case of severe hypoglycemia (24 mg/dL). The presence of signs was associated with hypoglycemia (OR = 8.1, CI 95% 2.6-25.1). The arterial hypertension persisted as a risk factor for the incidence of signs (OR = 3.6, CI 95% 1.2-11.3). Fasting glucose below 75 mg/dL increased the risk of hypoglycemia during the test (OR = 9.5, CI 95% 2.6-35.1).

**Conclusion:**

: In this study, high incidence of adverse effects during the OGTT was observed in post-bariatric surgery patients. If these results are confirmed by further studies, the indication and regulation of the OGTT procedure must be reviewed for these patients.

## INTRODUCTION

The oral glucose tolerance test (OGTT) is a time-honored test, which has been ordered since 1922 (1) with multiple indications in clinical practice. It is considered the gold standard for the screening and diagnosis of gestational diabetes, is used in the diagnosis of type 2 diabetes in conjunction with measurements of fasting glucose and glycated hemoglobin, and represents a valuable choice in the investigation of prediabetes (2,3). In addition, the OGTT is used in the risk assessment of cardiovascular disease (3,4). Despite its greater sensitivity than fasting glucose or glycated hemoglobin measurements in diagnosing type 2 diabetes (3,5,6), the use of the OGTT is less widespread for being a labored test and of low reproducibility.

The OGTT is a very safe procedure and is performed in most clinical laboratories in large cities. The adverse effects reported during the test are nausea and, occasionally, vomiting (7). However, most OGTT studies are not recent and fail to take into account the new clinical reality: the large number of indications of this test for patients who underwent bariatric surgery, both for the re-evaluation of the glycemic metabolism status after surgery (8) and for screening of gestational diabetes. It is known that the remission rates of type 2 diabetes after bariatric surgery orbit around 80% (9) and there occurs a significant improvement in fertility as well as in the polycystic ovary syndrome in women (10).

There is a worldwide upward tendency for obesity and in the number of bariatric operations (11). In Brazil, 48% of the female and 50.1% of the male and 16.9% of the female and 12.4% of the male over 20 years are, respectively, overweight and obese (12). Morbid obesity afflicts 3% of the population, with a significant number of candidates for the surgical treatment for this condition. From 2003 to 2010, the number of bariatric operations in Brazil nearly quadrupled – 16,000 to 60,000 (13).

As from 2005, a new clinical condition related to bariatric surgery began to be recognized, which was termed post-gastric bypass hyperinsulinemic hypoglycemia – a condition with repeated prandial episodes of severe neuroglycopenic hypoglycemia (11,14,15). The risk of severe hypoglycemia is two to seven times greater in individuals who underwent gastric bypass compared to non-bariatric patients or to those who only had restrictive gastric banding (11).

Another complication, more frequent than hyperinsulinemic hypoglycemia, occurring in patients after bariatric surgery is the dumping syndrome. This condition is found in 10-15% of the post-gastric bypass patients (16). Thus, the OGTT is used in the investigation of several clinical conditions after bariatric surgery.

Four studies used the OGTT in the evaluation of bariatric surgery patients with hypoglycemia, with different loads of dextrosol administered (some used 75 g dextrosol, others, 100 g) (9,14,17,18). None of these studies was intended to describe adverse effects that might be observed during the test; rather, their aim was to investigate the likely causes of hypoglycemia by measuring related parameters, such as blood glucose, insulin, C-peptide and glucagon-like peptide type 1 (GLP-1). Nearly all of those studies were conducted under medical supervision (9). Various authors who studied glycemic behavior in patients operated on by Roux-en-Y gastric bypass chose to use mixed meals, with lower carbohydrate concentrations than the standard dextrosol tests, probably to prevent the signs and symptoms of the dumping syndrome and reactive hypoglycemia, which can negatively impact an individual’s social life (19,20,21).

Since the severe episodes of hypoglycemia and dumping are triggered by food ingestion, the hypothesis of the present study was that a carbohydrate overload such as that given in the OGTT could cause a similar clinical picture. The objective of the present study was to investigate the incidence and risk factors for adverse effects to OGTT in post-bariatric surgery patients.

## SUBJECTS AND METHODS

The present study examines the incidence of adverse effects in 128 patients who underwent bariatric surgery (Roux-en-Y gastric bypass) and were referred for an OGTT at a clinical laboratory with ISO 9001:2008 certification and accreditation by the PALC/SBPC (Brazilian Society of Clinical Pathology) and DICQ/SBAC (Brazilian Society of Clinical Analyses) in Belo Horizonte, Brazil, from January 2010 to December 2011. All the participants presented to the laboratory spontaneously for the OGTT ordered by their assisting physician and accepted to participate in the present study (no patient declined).

The clinical data were collected by a single physician (HFAA) using a standardized questionnaire comprising sociodemographic (age, gender) and anthropometric (weight and height) variables, specialty of the ordering physician, morbid conditions reported, postoperative time of bariatric surgery, justification for the OGTT, and description of symptoms (dizziness, weakness, nausea, and diarrhea) and signs (tremor, tachycardia – heart rate ≥ 100 – and sweating). Hypoglycemia was defined as capillary blood glucose ≤ 50 mg/dL (14).

The participants were weighed wearing light clothing and barefoot; a Welmy scale (150 kg capacity) with accuracy of 100 g was used.

Arterial blood pressure (BP) was measured prior to the administration of dextrosol using a Becton Dickinson aneroid sphygmomanometer, with the patient in the supine position. The measurements were taken after a minimum resting period of 30 minutes, without consumption of caffeine or cigarettes for the same length of time, which was extended when symptoms occurred during the test.

The OGTT protocol consisted of the collection of a blood sample for glucose measurements after an 8 to 14-hour fast, according to manufacturer instructions (Gluc UP- Newprov^®^). The blood was collected into a tube containing anticoagulant EDTA and sodium fluoride (zero time). The serum glucose levels were measured by the hexokinase, Modular P^®^, Roche^®^ method. The coefficient of variation for glucose in that laboratory is 2.4% (mean, 82 mg/dL) and 2.6% (mean, 275 mg/dL).

A 75-g dose of dextrosol (75 g of anhydrous glucose in 300 mL of water) was administered orally for five minutes. The patients stayed seated and resting, under observation over the course of the test; another blood sample was taken from all patients for glucose levels at 120 minutes.

Capillary blood glucose levels of all patients were checked using a properly calibrated Accu-chek Advantage Johnson meter at baseline and every 30 minutes after the ingestion of dextrosol. If hypoglycemia symptoms occurred out of the established times for blood draw, supplementary capillary blood glucose measurements were performed and recorded. These capillary blood glucose measurements during the OGTT were conducted for the present study, yet they are not included in the routine OGTT protocol of the laboratory where the study was conducted. As a quality control laboratory procedure, a comparative study was performed between the measurements of venous and capillary glucose prior to the undertaking of the present study, including two groups of patients: 1) fasting glucose ≥ 60 mg/dL (group 1), and 2) glucose values < 60 mg/dL (group 2). Good correlations were found between the measurements. The median of venous blood glucose for group 1 was 81 mg/dL, and capillary, 92 mg/dL, with correlation of 0.825 (p < 0.0001). Group 2 had median venous blood glucose of 32 mg/dL, and capillary glucose of 43.5 mg/dL, with correlation of 0.879 (p < 0.0001).

All the study participants provided signed informed consent. The study protocol was approved by the Research Ethics Committee of the Universidade Federal of Minas Gerais, Belo Horizonte, Brazil (0385.0.203.000-11).

### Statistical analysis

The data were stored in an Excel^®^ software database and analyzed using STATA™ software version 10.0.

Descriptive analyses were performed for the frequency distribution of the variables and measures of central tendency (mean and median) and variability (standard deviation); mean and standard deviation were used for continuous variables with parametric distribution.

Pearson’s chi-square test was used for the categorical variables.

Logistic regression analysis was used in the evaluation of the relationship between dependent variables when p < 0.20 in the univariate analysis. Values of p < 0.05 were considered to be statistically significant.

## RESULTS

The great majority of the 128 patients were female; the mean age was 39 ± 11.8 years, and the median BMI, 30.4 kg/m^2^. Depression was the predominant morbidity reported, followed by arterial hypertension, hypothyroidism, dyslipidemia, and diabetes. Thirty-eight (29.7%) patients were pregnant (Table 1). The postoperative time of bariatric surgery ranged from 3 to 144 months, with a mean of 53 ± 34.0 months and median of 53 months (Table 1).

**Table 1:** Profile of the patients who underwent the OGTT between 2010-2011

		Hypoglycemia		Adverse signs	
	
		Yes	No	Yes	No
	n = 128				
Gender					
Female	117 (91.4%)	18	99	32	85
Male	11 (8.6%)	1	10	1	10
Age (years)					
< 40	83 (64.8%)	14	69	21	62
≥ 40	45 (35.2%)	5	40	12	33
Pregnant					
Yes	38 (29.7%)	2	36	10	28
No	90 (70.3%)	17	73	23	67
Reported morbidity					
Depression	24 (18.8%)	6	18	7	17
Hypertension	22 (17.2%)	4	18	10	12
Hypothyroidism	17 (13.3%)	4	13	7	10
Dyslipidemia	10 (7.8%)	1	9	3	7
Type 2 diabetes	3 (2.3%)	0	3	2	1
Postoperative time					
< 3 years	46 (35.9%)	5	41	12	34
≥ 3 years	82 (64.1%)	14	68	21	61

The reasons for ordering an OGTT were the presence of symptoms suggestive of hypoglycemia in 46 patients (35.9%); gestation in 38 cases (29.7%); health check-up for 14 patients (10.9%); elevated fasting glucose in eight patients (6.3%); type 2 diabetes prior to surgery in seven patients (5.5%); weight gain after surgery in six patients (4.7%), and eight patients were unaware of the reason for the request (6.3%).

The physicians who ordered the test were gynecologists (49, 38.3%); endocrinologists (22, 17.2%); general practitioners (22, 17.2%); surgeons (15, 11.7%); cardiologists (5, 3.9%); otolaryngologists (4, 3.1%), among other specialties (11, 8.6%).

Table 2 shows the mean capillary blood glucose values of the patients during the OGTT, fasted and then every 30 minutes up until 120 minutes.

**Table 2:** Capillary blood glucose values during the OGTT (n = 128)

	Mean ± Standard deviation	Median	Amplitude
Baseline glucose	86.4 ± 10.9	85	54–123
Glucose at 30 min	221.2 ± 48.8	218.5	84–389
Glucose at 60 min	183.6 ± 62.6	177.5	58–449
Glucose at 90 min	114.4 ± 47.4	109.5	33–252
Glucose at 120 min	75.1 ± 26.4	68	24–162

Eighty-three patients (64.8%) manifested adverse effects during the OGTT: nausea (49, 38.4%), dizziness (39, 30.5%), weakness (33, 25.8%), diarrhea (30, 23.4%), hypoglycemia (19, 14.8%), tachycardia (18, 14.1%), tremor (17, 13.3%), and sweating (16, 12.5%).

Among the study patients, 14.8% had two simultaneous adverse effects during the test, 13.3% manifested three adverse effects, and 16.4% showed four or more signs and symptoms during the test.

One patient had an episode of severe hypoglycemia (24 mg/dL) with tremor, nausea, dizziness, weakness and sweating. Two patients had very low blood glucose levels at 90 minutes and 120 minutes (33 and 24 mg/dL, respectively); a glucose level of 33 mg/dL was found in a woman pregnant with triplets.

On univariate analysis, patients with baseline (fasting) glucose below 75 mg/dL showed a greater risk for hypoglycemia (OR = 6.7, CI 95% 1.95-23.09). After adjusting for gender, age, postoperative time and arterial hypertension, this risk remained significant (Table 3).

**Table 3:** Multivariate analysis: association between hypoglycemia and patient characteristics (n = 128)

	Hypoglycemia		OR (CI 95%)*	p

	Yes	No		
Gender				
Female	18	99	2.34 (0.26-20.78)	0.45
Male	1	10	1.0	
Age (years)				
< 40	14	69	1.0	
≥ 40	5	40	0.63 (0.19-2.02)	0.43
Postoperative time				
< 3 years	5	41	1.0	
≥ 3 years	14	68	2.57 (0.77-8.50)	0.12
Fasting blood glucose				
≥ 75 mg/dL	12	103	1.0	0. 00
< 75 mg/dL	7	6	9.5 (2.57-35.11)	
History of arterial hypertension				
No	15	91	1.0	0.43
Yes	4	18	1.76 (0.43-7.24)	

* Adjusted for all the sociodemographic and clinical variables.

The presence of signs (tremor, tachycardia, sweating) was associated with hypoglycemia (OR = 8.14; CI 95% 2.63-25.12) after adjusting for gender, age, postoperative time and arterial hypertension (Table 4).

**Table 4:** Multivariate analysis of the risk factors for the incidence of adverse signs in the128 patients who underwent the OGTT

	Adverse signs		OR (CI 95%)*	p

	Yes	No		
Gender				
Female	32	85	3.52 (0.39-31.8)	0.26
Male	1	10	1.0	
Age (years)				
< 40	21	62	1.0	0.94
≥ 40	12	33	0.96 (0.36-2.59)	
Postoperative time				
< 3 years	12	34	1.0	0.40
≥ 3 year	21	61	0.67 (0.26-1.74)	
History of arterial hypertension				
No	23	83	1.0	0.03
Yes	10	12	3.64 (1.17-11.34)	
Hypoglycemia				
No	22	87	1.0	0.00
Yes	11	8	8.14 (2.63-25.12)	

* Adjusted for all sociodemographic and clinical variables.

Arterial hypertension posed greater risk for the incidence of signs (OR = 3.64; CI 95% 1.17-11.34) after adjusting for gender, age, postoperative time, and hypoglycemia (Table 4).

In the separate analysis of the women, patients with baseline (fasting) glucose below 75 mg/dL showed an increased risk for hypoglycemia (OR = 19.69; CI 95% 3.59-107.8), and the pregnant patients had a lower risk for hypoglycemia (OR = 0.17; CI 95% 0.03-0.84) after adjusting for age, preoperative time, and arterial hypertension (Table 5).

**Table 5:** Multivariate analysis: association between hypoglycemia and characteristics of the women in the study (n = 117)

	Hypoglycemia		OR (CI 95%)*	p

	Yes	No		
Pregnant				
Yes	2	36	0.17 (0.03-0.84)	0.03
No	16	63	1.0	
Age (years)				
< 40	14	59	1.0	0.10
≥ 40	4	40	0.35 (0.98-1.25)	
Postoperative time				
< 3 years	5	39	1.0	0.18
≥ 3 years	13	60	2.31 (0.67-7.92)	
Fasting blood glucose				
v≥ 75 mg/dL	11	93	1.0	
< 75 mg/dL	7	6	19.69 (3.59-107.8)	0.00
History of arterial hypertension				
No	15	82	1.0	0.60
Yes	3	17	1.44 (0.36-5.66)	

* Adjusted for all sociodemographic and clinical variables.

## DISCUSSION

The OGTT has a low incidence of adverse effects when administered to non-bariatric patients (7). However, the present study warns of the high incidence (64.8%) of adverse effects and hypoglycemia (14.8%) during the OGTT in post-bariatric surgery patients. Other studies have also pointed to that possibility, although it was not the focus of those studies. Roslin and cols. assessed the incidence of hypoglycemia in 36 patients at six postoperative months of Roux-en-Y gastric bypass or more; those authors used a test with 100 g dextrosol and hourly blood glucose and insulin measurements over the course of four hours. More than two-thirds (72%) of the patients showed hypoglycemia (blood glucose ≤ 60 mg/dL); the lowest glucose level found was 32 mg/dL (9).

Adverse effects such as flushing, palpitations, nausea, sweating and diarrhea were observed in all eight post-bariatric surgery patients in another study, in which the OGTT was performed with 100 g dextrosol administered prior to, and three months after, surgery with the aim of comparing the levels of glucose, insulin, vasoactive intestinal peptide and neurotensin at those two timepoints (18).

A recent study used the OGTT as a provocation test to evaluate the dumping syndrome in 25 patients who underwent sleeve gastrectomy. Symptoms suggestive of dumping occurred during the OGTT in 40% of the patients six months after the surgery and in 33% after 12 months. Hypoglycemia (blood glucose < 60 mg/dL) during the OGTT was noted in 33% of the patients both at 6 and 12 postoperative months (22).

One strength of the present study was the large number of patients evaluated. Regardless of the asymmetrical gender distribution, the fact that most patients were female may be due to the greater prevalence of class III obesity among women, resulting in a higher number of surgical procedures (13). The mean age of the study participants (39 years) was similar to that of bariatric patients in Brazil (41 years) (13).

Paradoxically, hypoglycemia, one of the major adverse effects found with the OGTT, was also the predominant reason for ordering the test by the attending physicians. It was noted that the dextrosol overload could replicate those symptoms, likely to a more severe degree than would a routine meal of the patients.

The second most frequent justification for ordering the OGTT was gestation (29.7%). It is known that the weight loss following bariatric surgery increases the fertility rate, both in men and women, for a number of reasons, and it is also an important factor of improvement in insulin sensitivity and polycystic ovary syndrome in women (10). Since the OGTT is recommended by the World Health Organization (WHO) and the American Diabetes Association (ADA) for gestational diabetes screening, it is a natural consequence that a large number of pregnant women who underwent bariatric surgery take this test.

Among the medical specialties of the physicians who ordered the OGTT, gynecologists were prominent, probably because of the great number of pregnant women in the study and the ensuing need for gestational diabetes screening. As expected, endocrinologists and general practitioners also predominated, likely in the search for potential hypoglycemia or other alterations that might arise postoperatively.

The increased risk of adverse signs in patients with a history of hypertension is independent of hypoglycemia, gender or age. It has been suggested that those patients are more sensitive to the BP reduction secondary to the dumping syndrome, thus manifesting symptoms earlier than normotensive patients by the passing of fluid from the vessels to the intestinal lumen, with a drop in circulating volume.

Baseline blood glucose of less than 75 mg/dL was a predictor of hypoglycemia during the test both for the overall group of patients and the women when evaluated separately.

Compared with the non-pregnant women, the pregnant patients showed a smaller likelihood of developing hypoglycemia during the test. This is probably due to the fact that the OGTT is typically ordered for gestational diabetes screening between week 24–28 of gestation, a period within which insulin resistance in already present as a result of the increased production of placental lactogen, in addition to the higher concentrations of cortisol, estrogen, progesterone and prolactin, which also decrease the sensitivity to insulin (23).

This study shows a few limitations. In the laboratory where the OGTT was performed, the instructions to the patients were that they should fast for 8–14 hours, according to the recommendations of the dextrosol manufacturer, and not for 10–16 hours as prescribed by the WHO; it is impossible to evaluate the impact of this difference in fasting time on the occurrence of adverse effects. Another limitation is that the information about arterial hypertension was self-reported by patients who knew they had been diagnosed with hypertension or were in use of some anti-hypertensive drug, not patients who showed elevated BP on the day of the OGTT. It is not possible to be sure of the operative technique employed either, since the “Roux-en-Y” gastric bypass information was also reported by the patients, and this could be due to the fact that the Roux-en-Y technique is currently the most commonly used (24,25) (nearly 80% of the surgical procedures for the treatment of obesity) (13). Even if the surgery was in fact Roux-en-Y gastric bypass, there are variations in the technique, e.g., the placement of the ring of containment around the pouch, size of the gastric pouch, among others, that could determine variations in the presence and intensity of the symptoms of dumping.

The findings of the present study demonstrate that the OGTT, when performed with patients who previously underwent bariatric surgery, entails risks for adverse effects, at least limiting, if not severe. The authors believe that this fact was not properly valued in other studies. The indication of OGTT should be reviewed for patients who underwent bariatric surgery, considering that it is not the only diagnostic choice for the assessment of glycemic states. Moreover, OGTT values of reference have not been established for this specific group of patients, which makes the use of this test even more questionable for the diagnosis of type 2 diabetes, gestational diabetes and evaluation of the states suggestive of hypoglycemia in post-bariatric surgery patients. It is imperative that clinical pathology laboratories provide adequate facilities and permanent medical supervision to manage potential clinical instability during the OGTT in post-bariatric surgery patients. Special attention should be given to hypertensive patients as well as to those who start the test with blood glucose levels of less than 75 mg/dL.
